# Comprehensive analysis of prognostic value and immune infiltration of CXC chemokines in pancreatic cancer

**DOI:** 10.1186/s12920-022-01246-4

**Published:** 2022-04-25

**Authors:** Yanhua Jing, Fengjiao Wang, Ke Zhang, Zhen Chen

**Affiliations:** 1 Department of Integrative Oncology, Fudan University Shanghai Cancer Center, Shanghai, 200032 China; 2 Department of Oncology, Shanghai Medical College, Fudan University, Shanghai, 200032 China

**Keywords:** Pancreatic cancer, CXC chemokines, Immune infiltration, Tumour microenvironment, CXCL5

## Abstract

**Background:**

The prognosis of pancreatic cancer is poor, with a 5-year survival rate of less than 10%. Studies have shown that chemokines in the tumour microenvironment are often altered, which is associated with immune infiltration and the prognosis and survival of pancreatic cancer patients.

**Methods:**

Multiomics and bioinformatics tools were used to clarify CXC chemokine expression and its role in the pancreatic ductal adenocarcinoma (PDAC) immune microenvironment.

**Results:**

Most CXC chemokines were upregulated in pancreatic cancer and correlated with patient prognosis. CXC chemokines can activate cancer-related signalling pathways and affect immune infiltration. Furthermore, most CXC chemokines were significantly correlated with the abundance of macrophages, neutrophils and dendritic cells. CXCL5 was selected as a hub gene, and a variety of immune checkpoints, including PD-1/PD-L1 and CTLA-4, were identified.

**Conclusion:**

Our study provides novel insights into CXC chemokine expression and its role in the PDAC immune microenvironment. These results can provide more data about prognostic biomarkers and therapeutic targets of PDAC.

## Background

Pancreatic ductal adenocarcinoma (PDAC) is a highly lethal malignant tumour and accounts for more than 85% of pancreatic cancers. In 2020, cancer statistics data showed that there were 60,430 new cases and 48,220 deaths related to pancreatic cancer [1]. Due to the difficulty in early diagnosis, up to 85% of patients are not eligible for surgical resection at diagnosis [2]. Over the past decade, advances in the diagnosis and treatment of advanced disease have made only modest incremental progress in patient outcomes. The survival time of patients has not been significantly prolonged, and the prognosis remains poor, with a 5-year survival rate of less than 10% [3]. These data illustrate the urgent need for novel and innovative treatments for PDAC.

The CXC family of chemokines (CXCL1 to CXCL17) are small (8 to 10 kDa) secreted proteins that are crucial for inflammation and antitumour immunity. These small proteins are secreted not only by tumour cells but also by leukocytes, fibroblasts, endothelial cells and epithelial cells, which can induce the directional migration of neutrophils and lymphocytes and regulate tumour-associated angiogenesis and cancer cell metastasis [1]. The tumour microenvironment (TME) is composed of tumour cells, immune cells and extracellular matrix. Studies on colorectal cancer have shown that chemokines in the TME are often altered, thus affecting tumour proliferation and metastasis [4, 5]. Li Yu and coauthors systematically analysed the expression and prognostic value of CXC chemokines in colorectal cancer and found that the expression of CXCL1, CXCL2, CXCL3, CXCL5 and CXCL8 increased, indicating that they can be used as potential therapeutic targets and prognostic factors and that these chemokines participate in the antitumour immune response [6]. Studies also show that CXC chemokines can lead to the promotion or inhibition of cancer, which mainly depends on the capacity to suppress or stimulate the action of the immune system, respectively [7]. However, the role of CXC chemokines in PDAC has not yet been clarified.

Therefore, in this study, we analysed the mRNA expression of the CXC chemokine family and its correlation with hallmarks of PDAC. We further clarified the correlation between CXC chemokines and immune cell infiltration in the TME of pancreatic cancer, which will help to better understand the CXC chemokine family and improve treatment designs and the accuracy of prognosis for patients with PDAC.

## Methods

### Expression analysis

We detected the mRNA expression of CXC chemokines in PDAC using three databases [8]. First, we explored CXC chemokine mRNA levels using ONCOMINE with the following thresholds: *P* value < 0.05, fold-change > 2, and a gene rank of top 10%. Then, in Gene Expression Profiling Interactive Analysis (GEPIA), we used a |Log2FC| cut-off of 1 and a *P* value cut-off of 0.05 to detect CXC chemokine expression levels. We further analysed the relative expression levels of CXC chemokines and their expression levels in different pathological stages and T stages.

### Survival analysis

In GEPIA, we evaluated the prognostic significance of CXC chemokines in PDAC using a Kaplan–Meier curve [9]. In the analysis, the median expression value of each CXC chemokine was used as a cut-off value to separate the samples into high/low expression groups. The Cox proportional hazards model was used to calculate the *P* value.

### Genetic alteration, coexpression, and interaction analyses

With cBioPortal, we analysed CXC chemokine genetic alterations in 295 pancreatic adenocarcinoma samples (The Cancer Genome Atlas [TCGA], Firehose Legacy and University of Texas Southwestern (UTSW) dataset, and Nature Communications). In the TCGA, we explored the potential coexpression of differentially expressed CXC chemokines. In STRING and GeneMANIA, the potential interactions of these CXC chemokines were detected.

### Cancer-related pathway and drug sensitivity analysis

In GSCALite, we detected cancer-related pathways and CXC chemokine drug sensitivity in PDAC samples [9]. In the cancer-related pathway analysis, high or low gene expression levels were dependent on median expression values, and pathway activity scores were defined by Student’s t test with FDR < 0.05. For the drug sensitivity analysis, we collected 265 small molecules from the Genomics of Drug Sensitivity in Cancer (GDSC) database, analysed their correlations with CXC chemokine expression, and calculated Pearson correlation coefficients.

### Enrichment analysis

Enrichment analysis of CXC chemokines in PDAC was conducted using Database for Annotation, Visualization, and Integrated Discovery (DAVID) 6.8, GEPIA, Metascape and GeneMANIA. First, GEPIA was used to extract the top five genes correlated with each CXC chemokine. Next, these correlated genes and CXC chemokines were used to explore the function of CXC chemokines in PDAC and to perform enrichment analysis (including gene ontology (GO), Kyoto Encyclopedia of Genes and Genomes (KEGG) pathway, and protein–protein interaction (PPI) network analysis). The analysis was conducted with the R programs “clusterProfiler” and “ggplot2” with *P* < 0.05. Then, we also submitted CXC chemokines and the correlated genes to Metascape to perform pathway and process enrichment with a minimum overlap of 3, *P* < 0.01 and a minimum enrichment of 1.5.

### Immune infiltration analysis

Immune infiltration analysis of CXC chemokines in PDAC was performed using the Tumor IMmune Estimation Resource (TIMER) and the TCGA. We evaluated the correlation between CXC chemokines and immune cells, immune score and stromal score using Spearman analysis. The analysis between CXC chemokines and the immune score and stromal score was used to estimate the algorithm.

### Correlation analysis of immune checkpoints

In view of the expression and prognostic implications of CXC chemokines in pancreatic cancer, we selected CXCL5 as the hub gene for the correlation analysis. The correlations between CXCL5 and 66 common immune checkpoints, including 23 immunoinhibitors and 43 immunostimulators, in the TCGA database were evaluated using Spearman analysis.

## Results

### CXC chemokine expression in PDAC

**Fig. 1:**
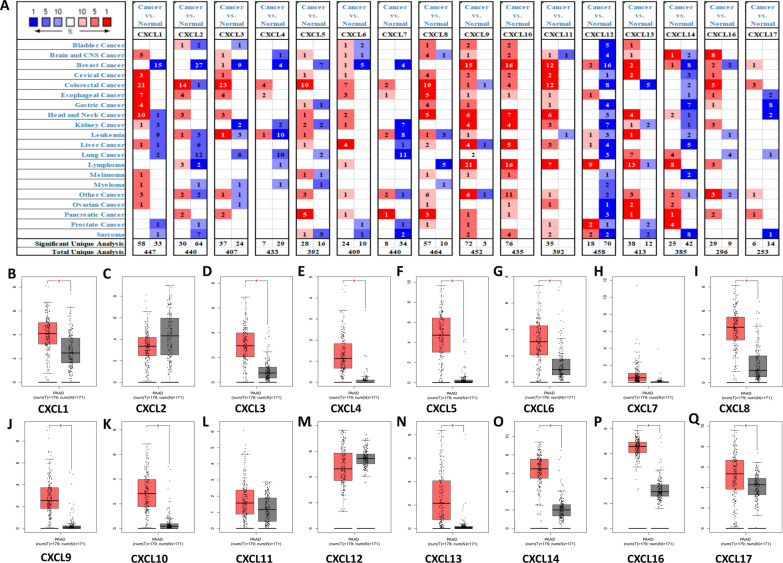
The mRNA levels of CXC chemokines in PDAC. Panel **A** shows the numbers of CXC chemokines with statistically significant mRNA upregulation (red) or downregulation (blue). **B**–**Q** Are the mRNA levels of CXC chemokines in PDAC tissues and normal tissues. *P < 0.05

**Fig. 2:**
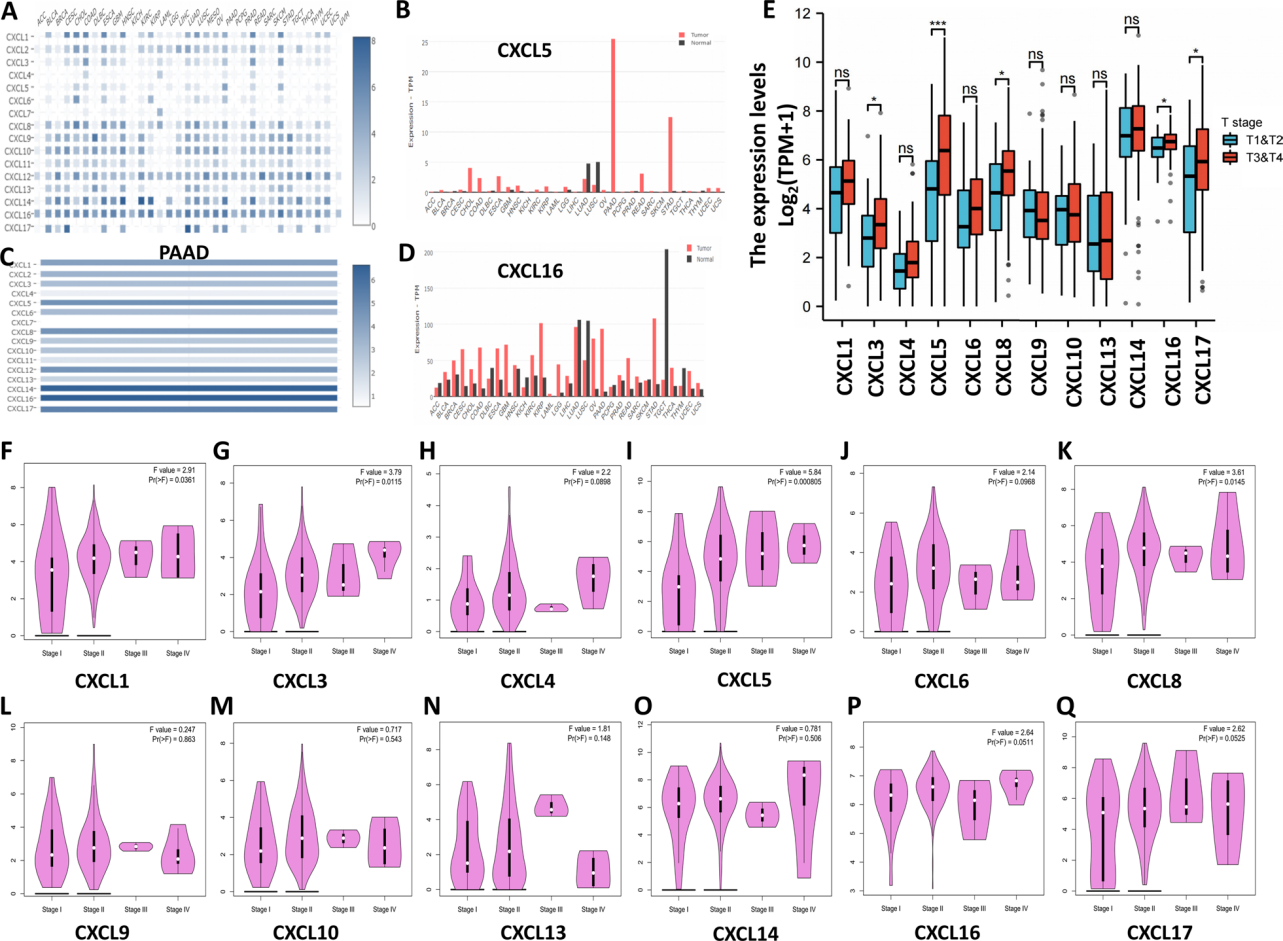
The mRNA levels of CXC chemokines. The relative levels of CXC chemokines (**A**), CXCL5 (**B**) and CXCL16 (**D**) in multiple cancer types and the relative levels of CXC chemokines in PDAC (**C**). The mRNA levels of CXC chemokines in different PDAC pathologic stages (**F**–**Q**) and T-staging (**E**)

We first explored the expression of 16 CXC chemokines in PDAC using ONCOMINE and GEPIA. ONCOMINE data showed that in addition to CXCL1, CXCL4, CXCL11 and CXCL12, the mRNA levels of the other 12 CXC chemokines were significantly increased in pancreatic cancer (Fig. 1A). CXCL2 exhibited the highest fold change (26.364), CXCL6 exhibited the lowest fold change (2.112) (Table 1). Using GEPIA, we found that the mRNA levels of 12 chemokines in PDAC, namely, CXCL1, CXCL3, CXCL4, CXCL5, CXCL6, CXCL8, CXCL9, CXCL10, CXCL13, CXCL14, CXCL16, and CXCL17, were significantly upregulated in pancreatic cancer tissues versus normal controls (Fig. 1B–Q). We further compared the relative expression levels of these 16 chemokines in various tumours and pancreatic cancer. The results showed that CXCL5 expression in pancreatic cancer was higher than that in other tumours, whereas in pancreatic cancer, CXCL16 expression was the highest (Fig. 2A–D).

**Table 1:** The mRNA levels of CXC chemokines in PDAC

CXC	Type	Fold change	*P value*	*t-*test	PMID
CXCL1	NA	NA	NA	NA	NA
CXCL2	Pancreatic ductal adenocarcinoma	26.364	0.001	5.416	16103885
CXCL3	Pancreatic carcinom	5.062	3.80E−7	5.806	19732725
CXCL4	NA	NA	NA	NA	NA
CXCL5	Pancreatic adenocarcinoma	5.697	6.38E−4	4.482	12750293
CXCL6	Pancreatic carcinoma	2.112	0.001	3.950	15867264
CXCL7	Pancreatic ductal adenocarcinoma	3.191	7.92E−5	4.139	16053509
CXCL8	Pancreatic carcinoma	4.573	1.71E−5	5.854	15867264
CXCL9	Pancreatic ductal adenocarcinoma	2.377	6.21E−4	4.782	16103885
CXCL10	Pancreatic ductal adenocarcinoma	3.950	1.41E−4	4.190	19732725
CXCL11/12	NA	NA	NA	NA	NA
CXCL13	Pancreatic ductal adenocarcinoma	7.592	0.004	3.085	15548371
CXCL14	Pancreatic adenocarcinoma	12.119	1.49E−5	6.699	12651607
CXCL16	Pancreatic adenocarcinoma	2.418	0.003	4.017	12651607
CXCL17	Pancreatic ductal adenocarcinoma	2.154	0.010	2.436	16053509

We identified CXC chemokine expression in PDAC tissues of different pathologic stages using GEPIA (Fig. 2F–Q). The results showed that the expression levels of CXCL1, CXCL3, CXCL5, and CXCL8 were related to disease stage, and the expression levels increased with disease progression. The TCGA database was also used to explore the correlation between PDAC T stage and CXC chemokine expression levels (Fig. 2E). The results showed that the expression levels of CXCL3, CXCL5, CXCL8, CXCL16 and CXCL17 were correlated with T stage, and the expression level increased with increasing T stage.

### Prognostic value of CXC chemokines in PDAC

**Fig. 3:**
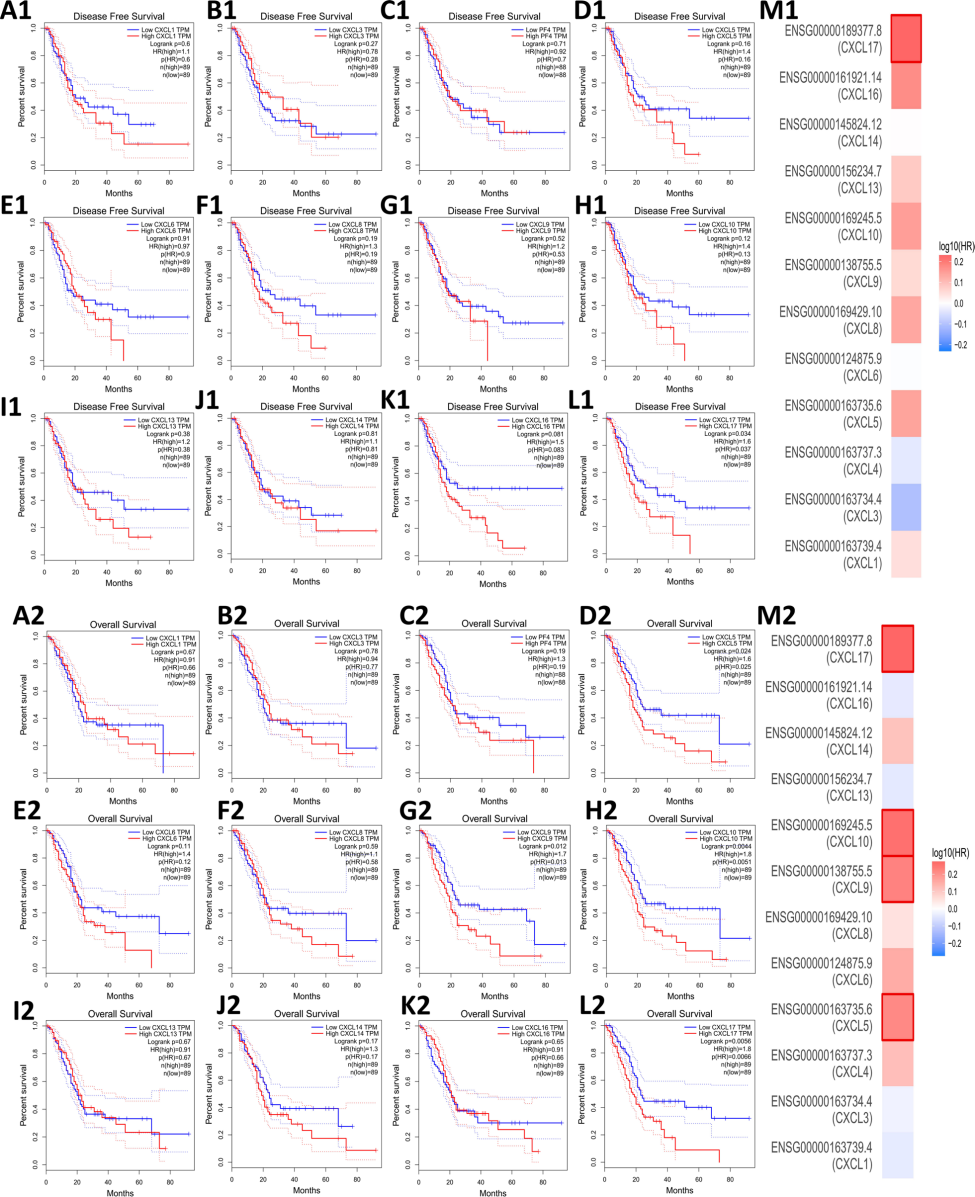
The disease-free survival (**A1**–**L1**) and overall survival (**A2**–**L2**) curves of CXC chemokines in PDAC. Estimation of the prognostic value of CXC chemokine genes in PDAC by using the Mantel–Cox test (**M1**, **2**)

We assessed the relationship between CXC chemokine expression and the survival (disease-free survival and overall survival) of PDAC patients using GEPIA. Based on data from GEPIA, we found that PDAC patients with high expression of CXCL17 (HR = 1.6, *P* = 0.037) had worse disease-free survival than those with low expression (Fig. 3A1–M1). Regarding overall survival, we found that PDAC patients with high expression of CXCL5 (HR = 1.6, *P* = 0.025), CXCL9 (HR = 1.7, *P* = 0.013), CXCL10 (HR = 1.8, *P* = 0.0051), and CXCL17 (HR = 1.8, *P* = 0.0066) had worse overall survival (Fig. 3A2–M2). Therefore, our results suggest that CXCL5, CXCL9, CXCL10, and CXCL17 may act as prognostic biomarkers in PDAC.

### Genetic alteration, coexpression, and interaction analyses of CXC chemokines in PDAC

**Fig. 4:**
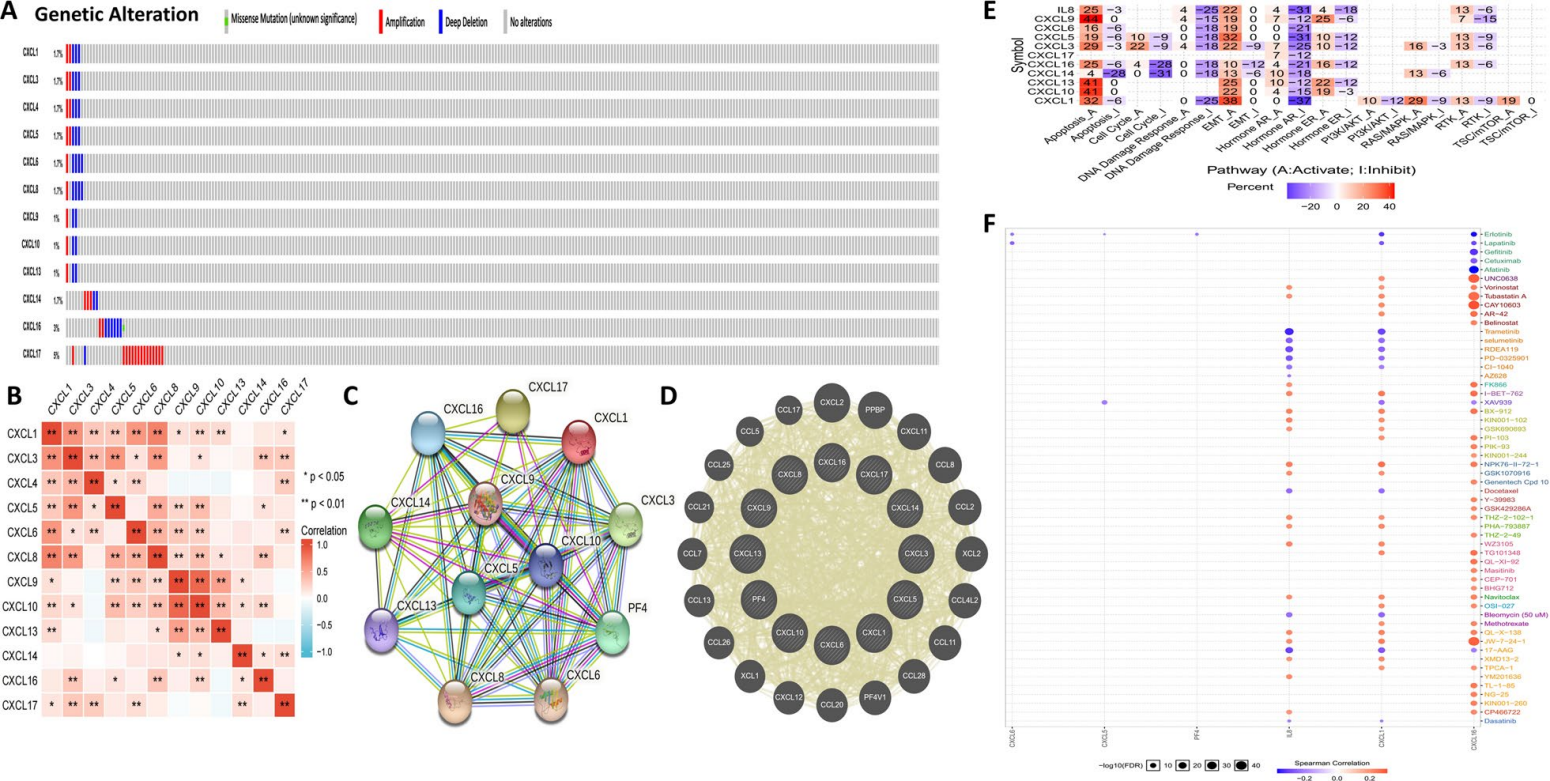
Mutations and interactions of differentially expressed CXC chemokines. Mutation of differentially expressed genes from cBioPortal (**A**). Correlation between each CXC chemokine in PDAC (**B**). A PPI network from STRING (**C**). Circular diagram of differentially expressed genes from GeneMANIA (**D**). The cancer-related pathways (**E**) and drug sensitivity analysis (**F**) of CXC chemokines in PDAC

We performed a comprehensive analysis of the molecular characteristics of differentially expressed CXC chemokines. First, we analysed the genetic alterations of these differentially expressed CXC chemokines using cBioPortal and found that CXCL1, CXCL3, CXCL4, CXCL5, CXCL6, CXCL8, CXCL9, CXCL10, CXCL13, CXCL14, CXCL16, and CXCL17 were altered in 1.7, 1.7, 1.7, 1.7, 1.7, 1.7, 1, 1, 1, 1.7, 3, and 5% of the queried PDAC samples, respectively. The most common change in these samples was gene amplification (Fig. 4A). Then, we used GEPIA to analyse the potential coexpression of differentially expressed CXC chemokines. There was a high correlation among the expression levels of CXCL1, CXCL5, CXCL6, CXCL8, CXCL9, and CXCL10, a moderate to high correlation among CXCL3, CXCL4, CXCL13, and CXCL16, and a low correlation between CXCL14 and CXCL17 (Fig. 4B). In addition, we conducted a PPI network analysis of differentially expressed CXC chemokines using Search Tool for the Retrieval of Interacting Genes/Proteins (STRING) and GeneMANIA to explore the potential interactions among them. The results of STRING analysis showed that the functions of CXC chemokines were mainly related to T cell and neutrophil chemotaxis and CXCR3 chemokine receptor binding with 12 nodes and 61 edges (Fig. 4C). The GeneMANIA results also showed that the functions of CXC chemokines were mainly related to cytokine activity, chemokine receptor binding and the cellular response to chemokines (Fig. 4D).

### Cancer-related pathway and drug sensitivity analysis of CXC chemokines in PDAC

We used GSCALite to analyse the function of CXC chemokines of PDAC in the famous cancer-related pathways (the TSC/mTOR, RTK, RAS/MAPK, PI3K/AKT, hormone ER, hormone AR, epithelial–mesenchymal transition (EMT), DNA damage response, cell cycle, and apoptosis pathways). The results showed that most CXC chemokines are related to the activation of apoptosis and the EMT pathway and the inhibition of the DNA damage response pathway and the hormone AR pathway (Fig. 4E). The drug sensitivity analysis revealed that the expression of CXCL1, CXCL8, and CXCL16 was positively correlated with drug resistance (Fig. 4F).

### Enrichment analysis of CXC chemokines in PDAC

**Fig. 5:**
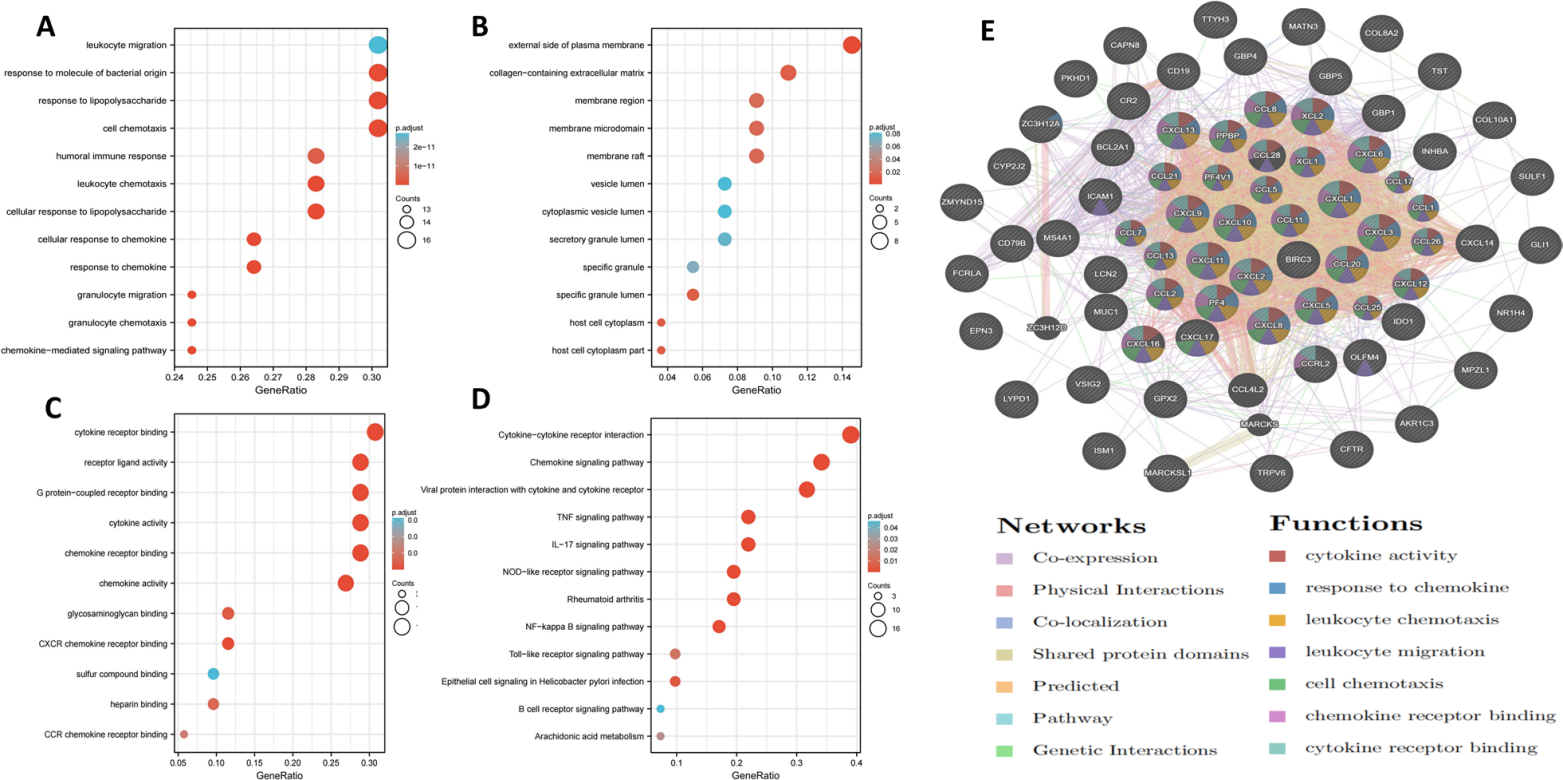
Enrichment analysis of CXC chemokines and neighbouring genes in PDAC. Bar plot of GO (**A**–**C**, including biological process terms, cellular component terms, and molecular function terms) and KEGG (**D**) enrichment and the PPI network (**E**)

**Fig. 6:**
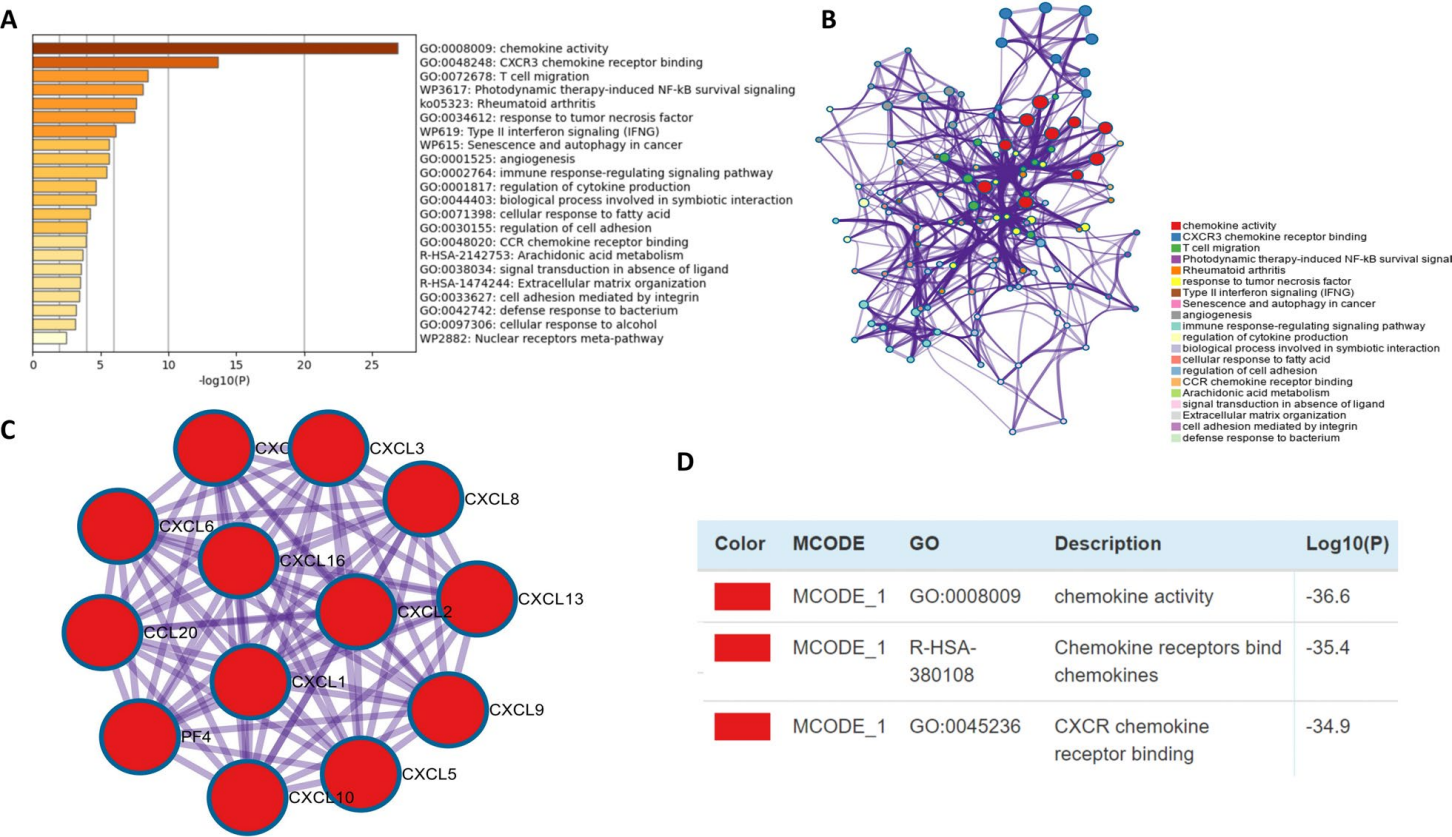
Enrichment analysis of CXC chemokines and neighbouring genes in PDAC. Bar graph of the top 20 enriched terms, coloured by *P* values (**A**). Network of enriched terms coloured by cluster ID (**B**). Protein–protein interaction network (**C**) and MCODE components (**D**) identified in CXC chemokines and neighbouring genes

Due to the vital function of CXC chemokines in PDAC, we performed GO function, KEGG pathway and PPI network enrichment analyses using DAVID 6.8, GEPIA, Metascape and GeneMANIA. First, GEPIA was used to obtain the top five correlated genes for each CXC chemokine (Table 2), and then CXC chemokines and the obtained genes were submitted to DAVID for enrichment analysis. GO functional enrichment analysis showed that CXC chemokines were mainly involved in cell chemotaxis, leukocyte migration, and cytokine and chemokine receptor binding (Fig. 5A–C). In KEGG pathway analysis, we found that CXC chemokines were enriched in cytokine–cytokine receptor interaction, the chemokine signalling pathway, the IL-17 signalling pathway, and the TNF signalling pathway (Fig. 5D). PPI network analysis showed that CXC chemokine function was mainly related to cytokine activity, binding with chemokine receptors, and the chemotaxis and migration of neutrophils, granulocytes, and leukocytes (Fig. 5E). To further validate the results, CXC chemokines and correlated genes were submitted to Metascape for GO function and KEGG pathway analysis. Similar to the previous results, the results revealed that CXC chemokines were mainly involved in chemokine activity, CXCR3 chemokine receptor binding, and T cell migration (Fig. 6A–C). Moreover, data from MCODE were extracted and suggested that CXC chemokines play roles in chemokine activity, chemokine receptor binding to chemokines, and CXCR3 chemokine receptor binding (Fig. 6D).

**Table 2:** The top 5 significant genes correlated with CXC chemokines in PDAC

CXC chemokines	Correlated genes
CXCL1	CXCL2, CXCL8, CXCL3, CXCL6, LCN2
CXCL3	CXCL2, CXCL1, ZC3H12A, CXCL8, CXCL5
CXCL4	CXCL3, TST, CYP2J2, GPX2, AKR1C3
CXCL5	BIRC3, CXCL3, CXCL8, BCL2A1, CCL20
CXCL6	LYPD1, NR1H4, PKHD1, TRPV6, CFTR
CXCL8	CXCL1, ICAM1, SULF1, CXCL2, INHBA
CXCL9	GBP5, CXCL10, GBP4, IDO1, CXCL11
CXCL10	CXCL11, CXCL9, GBP4, GBP5, GBP1
CXCL13	MS4A1, CD79B, CD19, CR2, FCRLA
CXCL14	COL8A2, GLI1, MATN3, COL10A1, ISM1
CXCL16	ZMYND15, MPZL1, MARCKSL1, CCRL2, TTYH3
CXCL17	VSIG2, MUC1, OLFM4, CAPN8, EPN3

### Analysis of the relationship between immune infiltration and CXC chemokines in PDAC

**Fig. 7:**
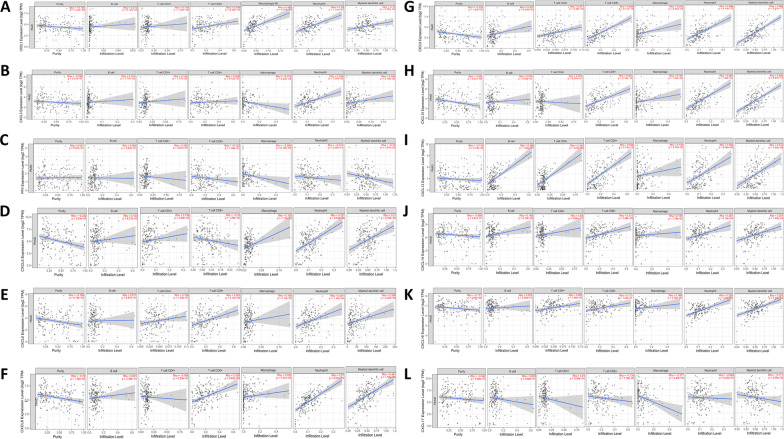
Analysis of the correlations between CXC chemokine expression and the immune infiltration levels of B cells, CD4+ T cells, CD8+ T cells, macrophages, neutrophils, and myeloid dendritic cells. And **A**–**L** are CXCL1-CXCL17, respectively

**Fig. 8:**
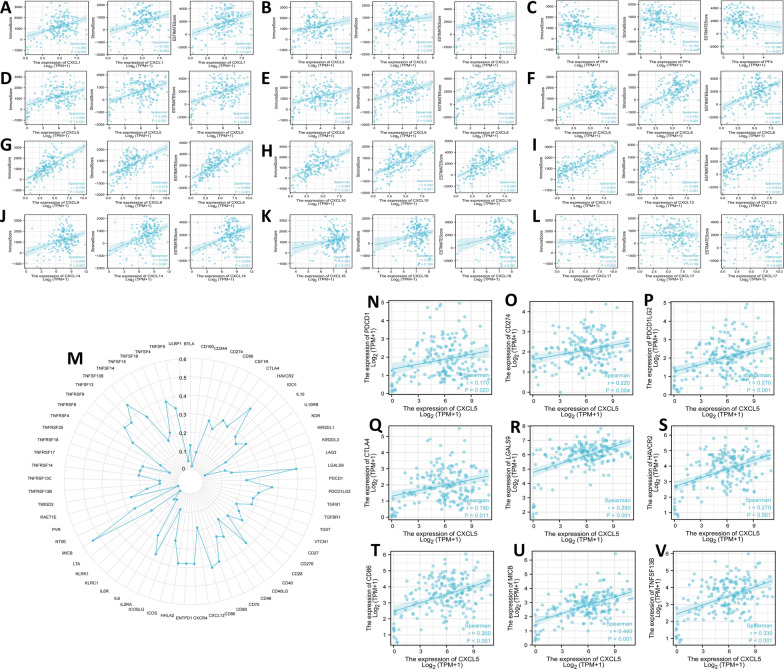
The correlations between CXC chemokines and immune score, stromal score, and ESTIMATE score (**A**–**L**). Correlation analysis of CXCL5 expression with immune checkpoint genes. **M** Correlation analysis of CXCL5 expression levels with over 60 common immune checkpoint gene levels in PDAC. **N**–**V** CXCL5 expression was positively closely related to PD-1, PD-L1, PD-L2, CTLA4, LGALS9, HAVCR2, CD86, MICB and TNFSF13B in PDAC

According to the TIMER results, most CXC chemokines show a significant correlation with the abundance of macrophages, neutrophils and dendritic cells (Fig. 7). With the exception of CXCL3, CXCL4, CXCL16, and CXCL17, all the CXC chemokines were significantly correlated with the abundance of CD8+ cells. Except for CXCL4, CXCL16 and CXCL17, the CXC chemokines were significantly correlated with the immune score. Except for CXCL3, CXCL16 and CXCL17, the CXC chemokines were significantly correlated with stromal score (Fig. 8A–L).

### Correlation analysis of immune checkpoints in PDAC

There were 5 immune checkpoints with correlation coefficients greater than 0.4: MICB, TNFSF13B, HAVCR2, LGALS9, and CD86; other immune checkpoints also had a certain correlation with CXCL5 (Fig. 8M). We also analysed the correlation of CXCL5 with CD274, PDCD1, PDCD1LG2, and CTLA4. These nine immune checkpoints were all significantly correlated with CXCL5 (Fig. 8N–V).

## Discussion

The incidence and mortality rate of pancreatic cancer is high, with the incidence rate ranking tenth in men and eighth in women and the mortality rate ranking fourth in both sexes in 2021 [1]. The TME is a local microenvironment that regulates tumorigenesis and tumour development and is closely associated with drug resistance, immune escape and prognosis. Previous studies have shown that CXC chemokines are upregulated in many tumours, can serve as prognostic biomarkers, and play a vital part in the interaction between the TME and cancer cells [10]. Therefore, clarification of CXC chemokine expression in PDAC and its correlation with cancer hallmarks is of great importance.

In this study, we used multiomics and various bioinformatics tools to elucidate the role of CXC chemokines in PDAC. Basically, all CXC chemokines were upregulated in PDAC, which correlated with patient prognosis. We also revealed that CXC chemokines can activate cancer-related signalling pathways and affect immune infiltration and are associated with drug resistance. In addition, CXCL5 was selected as a hub gene, and immune checkpoints of CXCL5 were identified.

We first explored the expression and prognostic value of CXC chemokines in PDAC. The results revealed that most CXC chemokines were upregulated in PDAC, and CXC chemokine expression increased with the progression of disease and T stage. Additionally, PDAC patients with high CXCL5, CXCL9, CXCL10 and CXCL17 levels had a poor prognosis, indicating that these 4 chemokines are prognostic biomarkers in PDAC. Additionally, previous studies have shown that the transcription level of CXC chemokine is increased in many tumours and can be used as a biomarker for different types of tumours. In breast cancer, CXCL1-2 can be used as a therapeutic target and biomarker, and its expression level is negatively correlated with the survival and prognosis of patients [11]. In cervical cancer, the transcription level of CXCL1/3/5/6/8/9/10/11/13/16/17 is significantly increased, which is associated with poor prognosis [12]. In prostate cancer, high CXCL16 and CXCR6 expression is an independent predictor of poor clinical prognosis [13]. In addition, Yang et al. showed that CXCL14 can inhibit tumour growth and play a multifunctional regulatory role in tumour progression [14]. Ala Litman and coworkers explained the importance of the CXCL8-CXCR2 axis in pancreatic cancer, which indicate that the serum chemokine CXCL8 is a better diagnostic and predictive marker than CXCR2, C-reactive protein, classic CA 19-9, and CEA protein [15]. Our results further confirmed these previous findings. Therefore, CXC chemokines likely play an important role in PDAC.

The alterations in the expression of CXC chemokines are related to the regulation of common cancer-related pathways. Our results highlight the stimulatory effects of CXC chemokines on apoptosis and the EMT pathway, as well as inhibition of the DNA damage response and the hormone AR pathway. The upregulated CXC chemokines may inhibit the DNA damage response and activate EMT, thus promoting tumorigenesis and progression. The CXCL-CXCR axis, namely, the binding of CXC chemokines with their corresponding ligands, can promote tumour invasion and metastasis in a variety of tumour types. In thyroid carcinoma, the CXCL12-CXCR4 axis can facilitate tumour cell migration, invasion and EMT [16]. In breast cancer, CXCL1 can elicit cancer progression and immune escape programs, and targeting the CXCL1/CXCR2 axis can improve treatment [17]. In pancreatic cancer, cancer cells and cancer-associated fibroblasts (CAFs) can interact through the CXCL-CXCR2 axis, thus promoting invasion and metastasis [18]. Nab‐paclitaxel can block pancreatic cancer cell migration and invasion effects by increasing CXCL10 expression in cancer cells and inhibiting IL-6 expression in CAF cells [19]. Necroptosis of pancreatic cancer cells at the invasion front can promote migration and invasion by releasing CXCL5 [20]. Additionally, in colorectal cancer, Zhang et al. demonstrated that the upregulation of CXCL1, CXCL2, and CXCL4 expression was associated with GNA13 overexpression [21]. Our findings further validate findings from previous studies.

Another important finding is that most CXC chemokines are significantly correlated with the abundance of macrophages, neutrophils, dendritic cells and CD4+/CD8+ T cells. As important components of the TME, both CXC chemokines and immune cells are involved in the regulation of tumour development, invasion and metastasis. The expression of chemokines can affect immune cell distribution in the TME, thus regulating the immune response. Different CXC chemokines affect different types of immune cells, resulting in immune activation or inhibition, depending on the cell type secreting CXC chemokines. CXCL1 and CXCL2 produced by tumour cells can promote the generation of monocytic myeloid-derived suppressor cells [22]. Pancreatic stellate cells can increase CXCL12 expression via the NF-kB pathway, thus promoting tumour growth and preventing cytotoxic T cells from infiltrating the tumour and killing cancer cells [23]. Follicular helper T cells can promote an immunoactive pancreatic cancer microenvironment by secreting CXCL13 and IL-21 [24]. Due to a significant correlation between CXC chemokines and immune cells, we hypothesize that some CXC chemokines may serve as important factors leading to immunosuppression in pancreatic cancer [25]. Furthermore, more experiments should be performed.

We also found that the expression of CXCL5 in PDAC was significantly correlated with that of many immune checkpoints, including PD-1/PD-L1 and CTLA-4. Plasma cytokines IL-18 and CXCL10 could indicate the anti-PD-1/PD-L1 treatment response in lung cancer patients and play an important role in selecting patients benefiting from PD-1/PD-L1 inhibitors [26]. In gastric cancer, tumour-associated macrophages autonomously express PD-L1 by secreting CXCL8, thus promoting immune evasion [27]. In pancreatic cancer, the inhibition of CXCL12 derived from carcinoma-associated fibroblasts could induce rapid T cell accumulation among cancer cells and play a synergistic role with anti-PD-L1 to kill cancer cells [28]. IFN-γ could enhance the efficacy of anti-PD-1 treatment by preventing the trafficking of CXCR2^+^CD68^+^ macrophages by blocking the CXCL8-CXCR2 axis [29]. Therefore, targeting CXC chemokines may be a therapeutic option to improve the efficacy of immune checkpoint inhibitors in pancreatic cancer.

At present, some clinical trials on the combined application of anti CXC or anti CXC receptor drugs are underway. Anti-CXCL12 (NOX-A12) combined with pembrolizumab can prolong the time on trial treatment compared with their last standard treatment and activate the Th1 immune response in patients with metastatic colorectal and pancreatic cancer [30]. A CXCR4 antagonist (BL-8040) combined with pembrolizumab and chemotherapy can increase the disease control rate of pancreatic cancer patients, promote the tumour infiltration of CD8+ T cells, and decrease the levels of myeloid-derived suppressor cells and circulating regulatory T cells [31]. A CXCR4 inhibitor (Plerixafor) combined with concurrent chemoradiation improved the local control of tumour recurrence in glioblastoma patients [32]. A CXCR4 antagonist (cyclam monomer) combined with a CD44 inhibitor (Star miR-34a) demonstrated antitumour and antimetastatic efficacy in breast cancer [33]. In hepatocellular carcinoma, a CXCR4 antagonist (BPRCX807) combined with sorafenib or anti-PD-1 produced synergistic antitumour effects, extended survival and suppressed distant metastasis [34].

In addition, many studies have revealed that certain CXC chemokines play an important role in many cancers, along with other diseases. For example, CXCL2 is related to bone erosion in rheumatoid arthritis [35]. CXCL14 was found to be a new biomarker for obesity, type-2 diabetes and liver fibrosis [36, 37]. Moreover, CXCL12 could be an indicator of bladder cancer microenvironment modulation [38]. High CXCL9 and CXCL13 levels conferred an improved prognosis in early breast cancer [39]. The levels of CXCL10 and CCL21 were associated with pain in pancreatic cancer patients [40]. Our research also has certain limitations. First, the relationship between CXC chemokine expression and clinicopathological features has not yet been clarified. The CXC chemokine analysis in our study was based on the mRNA level, which may not reflect changes in the protein level. Moreover, our results could be challenged with in vitro or in vivo experiments.

## Conclusion

Overall, our study provides novel insights into CXC chemokine expression and its role in the PDAC immune microenvironment. These results may provide more data about prognostic biomarkers and therapeutic targets for PDAC.

## Data Availability

The results were analysed online and aggregated directly from multiple databases without relevant accession numbers. Direct web links of datasets: ONCOMINE, https://www.oncomine.org/resource/login.html; Gene Expression Profiling Interactive Analysis (GEPIA, http://timer.comp-genomics.org/; TISIDB, http://cis.hku.hk/TISIDB/index.php; STRING, https://string-db.org/cgi/.

## Abbreviations

TME: Tumour microenvironment
PDAC: Pancreatic ductal adenocarcinoma
GEPIA: Gene Expression Profiling Interactive Analysis
TCGA: The Cancer Genome Atlas
GO: Gene ontology
BP: Biological process
CC: Cellular component
MF: Molecular function
KEGG: Kyoto Encyclopedia of Genes and Genomes
